# Semen analysis of subfertility caused by testicular carcinoma

**DOI:** 10.18502/ijrm.v13i7.7371

**Published:** 2020-07-22

**Authors:** Behrooz Ghasemi, Alimohammad Mosadegh Mehrjardi, Carolyn Jones, Nasrin Ghasemi

**Affiliations:** ^1^Maternal and Fetal Health Research Centre, Division of Developmental Biology and Medicine, Reproductive Medicine, School of Medical Sciences, St Mary's Hospital, University of Manchester, Manchester, UK.; ^2^Department of Traditional Pharmacy, Faculty of Traditional Medicine, Tehran University of Medical Sciences, Tehran, Iran.; ^3^Division of Developmental Biology and Medicine, Maternal and Fetal Health Research Centre, School of Medical Sciences, Faculty of Biology, Medicine and Health, University of Manchester, Central Manchester University Hospital NHS Foundation Trust, Manchester Academic Health Sciences Centre, St Mary's Hospital Manchester, UK.; ^4^Abortion Research Centre, Reproductive Sciences Institute, Shahid Sadoughi University of Medical Science, Yazd, Iran.

**Keywords:** Infertility, Testicular cancer, Semen analyses, Spermatogenesis.

## Abstract

**Background:**

Infertility is a common problem in testicular cancer. Affected men often decide to undergo sperm banking before chemo/radiotherapy. The cumulative effects of therapy can considerably reduce fertility.

**Objective:**

Testicular cancers impair fertilizing ability, even before diagnosis. This study tries to verify individual traits and semen quality in patients with testicular cancer.

**Materials and Methods:**

This observational study analyzed 190 semen of patients with testicular cancer (16 to 47 yr old) referred to the sub-fertility laboratory at the St. Mary hospital for semen banking prior to treatment carcinoma. Several aspects of their semen analyses were examined. The cases were divided into four different categories: seminoma, teratoma, mixed germ cell tumors and others.

**Results:**

The results showed that 23 cases were azoospermic, and 13 of the patients who were not azoospermic, their sperm of “normal” morphology were too few to count. Among patients that could produce spermatozoa, 59.4% had a sperm concentration of < 20 × 10${}^{6}$/ml. The mean of “motility excellent” and “sluggish” taken together in all the cases was 47.2%. More than 92% of the patients had an abnormal morphology. The morphology of sperm is the most sensitive semen parameter that is affected by testicular carcinoma.

**Conclusion:**

Abnormal spermatogenesis is seen in most patients with testicular cancer before treatment with radiation, chemotherapy, or surgery. The causes of poor semen quality in cancer patients are not well-understood, but the patients with impaired spermatogenesis should have precise examination to find out the correct diagnosis of problem and preserve the fertility before any treatment.

## 1. Introduction 

Testicular cancer is often cited as the most common cancer of young men and boys between 15 and 35 years of age (1). It is a relatively rare tumor, comprising only 1% of all malignant neoplasms in men (2). The statistics reviews across Europe and the United States show that it is increasing in incidence in Caucasian men (3-4). An unexplained rise in the occurrence of testicular cancer has been observed in the United States, with a 100% increase in the number of reported cases since 1936 (2). A similar trend has also been reported in several northern European countries. There is extensive geographical variation and the incidence rate of testicular cancer can fluctuate between countries (5).

Testicular tumors can be categorized into germ cell and non-germ cell tumors. Germ cell tumors arise from spermatogenic cells and comprise 95% of testicular neoplasms. Only 10% of cases of these tumors are malignant. Non-primary tumors such as lymphoma, leukemia, and metastases can also be presented as testicular masses (6).

Testicular cancers (TC) impair fertilizing ability, even before diagnosis (7). They affect the hypothalamic-pituitary-gonadal axis and consequently disturb spermatogenesis (8). These deleterious effects are dependent on the stage and type of seminoma, resulting in poor semen quality or even azoospermia (9). In many TC patients, sperm quality is already abnormal and may even lack viable spermatozoa at the time of diagnosis (10). The treatment for this type of cancer, usually performed by surgery, chemotherapy, or radiotherapy, further affects semen quality (9) and hormonal function (11), thus highly impairing male fertility. In fact, after cancer therapy, patients may become temporarily or permanently infertile (12). For that reason, it is strongly recommended that men diagnosed with TC undergo sperm banking to increase the probability of fatherhood in the future.

Semen analysis is a preliminary assessment of male infertility and can be done for several reasons such as unexplained infertility, screening sperm donors, examination of a male partner prior to reversal of female sterilization, post-vasectomy reversal, or assessment of patients banking semen before undergoing chemo/radiotherapy. In these patients, the preservation of male fertility is usually done through cryopreservation. This procedure stabilizes the cells at cryogenic temperatures, which is known as a useful aspect of cryobiology or continuation of life at low temperatures (13).

Previous study has not yet recognized whether TC histology may clarify different alterations to semen quality. Some researchers show that a nonseminoma usually effects more negatively on semen quality than a seminoma, but others fail to prove this difference (14).

While much has been progressed in the treatment and diagnosis of TC, knowledge about these patients is still needed to better understand their current lifestyles and future decision. Therefore, this is important to examine carefully men with impaired semen analysis.

Therefore, this study sets out to prove individual characters and semen quality in patients with testicular cancer, and to compare semen quality in them.

## 2. Materials and Methods

### Sample collection and delivery

In the current observational study, 190 semen samples from men with TC were collected after a minimum of 48 hr, but not longer than seven days of sexual abstinence. Patients suffering from systemic disorders like diabetes, hypertension, etc. were excluded from the study. To diminish the variability of semen analysis results, the number of days of sexual abstinence was kept constant as possible. Ideally, the specimen was passed in a private room close to the laboratory or it was delivered to the laboratory within 1 hr of collection.

Semen specimens were passed through masturbation and ejaculated directly into a 60 ml jar made of glass or plastic. It was warm, and kept at room temperature (25°C) to avoid reduction in sperm motility. All products were assessed for the absence of spermicidal properties prior to use.

### Initial examination

According to the WHO protocol, fresh specimens passed on the premises were placed in an incubator at 37°C until complete liquefaction had taken place. A normal semen sample liquefies within 60 min at room temperature, although usually this occurs within 15 min.

The semen sample was examined immediately after liquefaction or within 1 hr of ejaculation, first by simple inspection at room temperature.

The viscosity, sometimes referred to as consistency, of the liquefied sample was recognized as being different from coagulation. The pH was measured at a uniform time within 1 hr of ejaculation.

During the initial microscopic investigation of the sample, estimates were made of the concentration, motility, agglutination of spermatozoa and presence of cellular elements other than spermatozoa.

### Preparation for routine semen analysis

The volume of semen and the dimensions of the coverslip were standardized so that the analyses were always carried out in a preparation of a fixed depth of about 20 μm. A fixed volume of 10 μl semen was delivered onto a clean glass slide with a positive displacement pipette and covered with a 22 × 22 mm coverslip. The freshly made wet preparation was left to stabilize for approximately 1 min. Since sperm motility and velocity are highly dependent on temperature, the assessment of motility is preferably performed at 37°C, using a warmed stage.

At least five microscopic fields were systematically scanned until the motility of 200 sperm had been graded.

The length of a normal sperm head is defined as 4-5 μm, and for the purposes of motility assessment, sperm moving progressively at more than 5 head lengths/second can be defined as grade a. The count of 200 spermatozoa was repeated on a separate 10 μl specimen from the same semen sample and the percentages in each motility grade from the two independent counts were compared.

Each group of patients were compared with each of the rest using the “Independent sample test” and “Analysis of variance” was used to determine the significance of the differences between all the groups. The way in which the mean value of a variable was affected by the classification of the data could be determined by analysis of variance. The one-way analysis of variance is a generalization of the independent sample test (for the comparison of the means of two groups of data), and is appropriate for any number of groups. Rather than examining the difference between the means directly, analysis of variance looks at the variability of the data.

### Ethical consideration

The project was approved by the ethical committee of Manchester University (ref 03238).

## 3. Results 

In the semen analyses, samples were obtained from 190 patients that were referred to the sub-fertility department of St. Mary hospital, UK, with diagnoses of testicular cancer.

The different types of testicular carcinoma in 190 patients consisted as 19 mixed germ cell tumour cases (10%), 88 Seminoma cases (46.3%), 58 Teratoma cases (30.5%), and 25 other types of tumour cases (13.2%).

The patients could be categorized by their pathological diagnosis into four groups, seminoma, teratoma, mixed germ cell tumor, and other types of tumor (i.e. embryonal carcinoma, yolk sac tumor, etc.)

**Figure 1:**
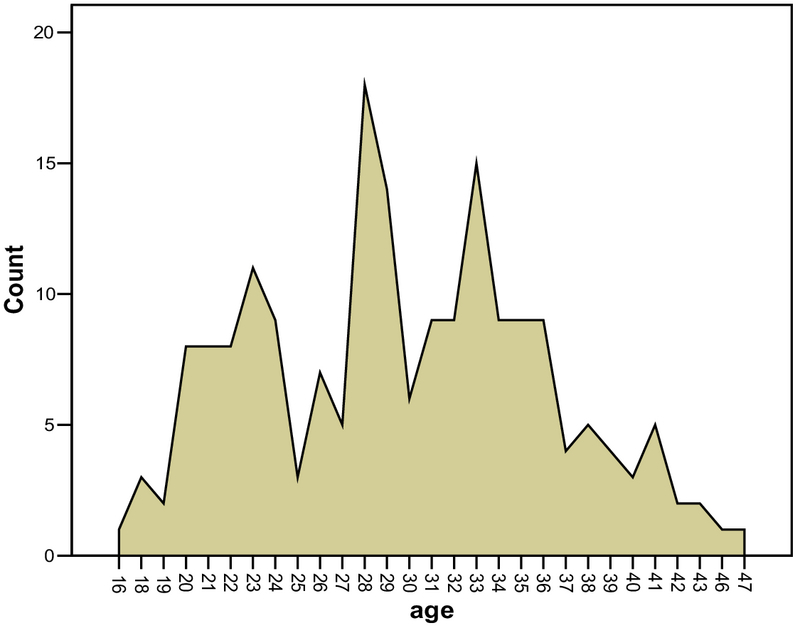
Distribution of patients by their age in testicular cancer (count *10${}^{6}$).

The age range of the volunteers was between 21-40 yr, and the mean age was 31.38 yr. While, the age range of the patients was 16-47 yr with the mean age as 29.75 yr and the median was 29 yr (Figure 1).

Patients diagnosed with mixed germ cell tumor were in the bracket of 18-40 yr. Patients in the seminoma group were in the 20-47 yr, while patients in the teratoma group were in the bracket of 16-41 yr. The mean age in the teratoma group was 26.5 yr. The patients whose carcinoma was categorized as `other types of tumor' were in a group with an age range of 18-41 yr, with the mean age being 29.80 yr.

The collected data for all the patients show that in 102 cases, information for the side of the tumor was missing, and there is not enough evidence to support a subsequent determination on to the side upon which testes occupied by the tumor lay. Among the rest of the patients, in 42 cases the tumor was of the left testis, and in 46 of them, the tumor was of the right testis. Table I shows the sidedness of the tumors in different types of testicular cancer.

The mean volume of the semen samples for the volunteers was 3.81 ml (range 2.5-8.5 ml). The mean volume for the whole group of patients was 2.6 ml. The lowest volume among all the patients was 0.3 ml and the highest volume was 10 ml. The lowest volume, which is 0.3 ml, belonged to one of the patient in the seminoma group and the highest volume (10 ml) belonged to a patient in the group diagnosed with mixed germ cell tumor.

Sixty patients with various type of testicular cancer had subnormal semen volume compared to the WHO (1999) reference range: 5 cases with mixed germ cell tumor (8%), 31 with seminoma cases (52%), 15 teratoma (25%), and 9 other types of tumor (15%).

The mean of the sperm population in volunteers was 111.5×10${}^{6}$/ml. Out of the 190 semen samples derived from the patients from different groups, 10 samples had no sperm concentration at all. The sperm count in these patients was nil and they were considered to have azoospermia.

The mean sperm concentration of all cases with testicular cancer was 24.7 × 10${}^{6}$/ml without azoospermic patients.

The minimum sperm count in those patients, who were producing spermatozoa was 0.09 × 10${}^{6}$/ml and the maximum sperm count was 167 × 10${}^{6}$/ml; 73 patients of the total had a normal sperm concentration with a mean sperm count of 49.3 × 10${}^{6}$/ml and 117 patients had an abnormal sperm concentration with a mean of 7.26 × 10${}^{6}$/ml. Table II summarizes the sperm concentration in various types of testicular cancer.

In the group containing volunteers, the mean of “motility excellent” was 35.2%, with a minimum of 25% and a maximum of 50%. All of the volunteers had 25% or more “motility excellent”. Also, in the same group, the mean of “motility excellent” and “motility sluggish” together was 65.8%. The immotile sperm had a mean of 25.4%, with minimum and maximum percentages of 15% and 40%, respectively.

The comparison of the results in cases with the reference range that is recommended by WHO showed that only 10 patients (52.6% of the total) had a normal motility. The mean of the “excellent motility” and “excellent motility” and “sluggish motility” together in this group was 49.9% and 62.4%, respectively.

In the seminoma group, the mean of excellent motility was 33.41%. Of this group, seven patients (8%) had no spermatozoa with “excellent” motility and 30.7% of all the patients had an “excellent” motility of < 25%. In the seminoma group, 41 patients (46.59%) had a normal motility with a mean of 50.49% for “excellent motility” and 62.54% for “motility excellent” and “sluggish” together.

The mean of “excellent motility” in the teratoma group was 34.67% with two patients having no spermatozoa with “excellent” motility. In this group, 25.9% of the patients had an “excellent” motility of < 25%, and 46.6% of them had “motility excellent” and “sluggish” together of < 50%.

In the group of patients with diagnoses of other types of testicular cancer, the mean of “excellent” motility was 34.44%, and three patients in this group (12% of the total in this group) had no spermatozoa with “excellent” motility.

Table III shows the mean of motility sluggish, motility non-progressive, and motility immotile in various types of testicular cancer.

In the group of volunteers, the normal morphology of sperm started from 50% and rose to 72%. Therefore, all of the cases had normal morphology of > 15% and the mean of normal morphology in this group was 65.7%. In the group of patients, 23 cases had morphology equal to 0 as 10 of them were azoospermic patients (i.e., without any production of spermatozoa) and in a further 13 patients, who were not azoospermic, their sperm of “normal” morphology were too few to count. Therefore, including all of these cases, the mean morphology for the patients was 6.55% with a maximum of 36%. In the whole group of patients, only 15 had a normal morphology which was 15% or more. More than 92% of the patients had an abnormal morphology. The mean of morphology in the patients with “normal morphology” was 18.07% and the mean for the patients with “abnormal morphology” was 5.57%.

In the teratoma group, among the non-azoospermic patients, the mean of morphology was 6.64% and > 91% of the patients had an “abnormal” morphology. In the group of patients diagnosed with mixed germ cell tumor, the mean of morphology was 8.5% and 77.8% of the patients with “abnormal” morphology. The mean of morphology in the patients diagnosed with other types of carcinoma was 5.23%, of which three patients, equal to 13.6% of the patients in this group, had too few normal sperm to count. Also, all of the patients in this group had “abnormal” sperm morphology.

**Table 1:** The sidedness of the tumors in different types of testicular cancer


**Diagnosis**	**Number of cases**	**Missing information**	**Left testis**	**Right testis**
**Mixed germ cell tumor**	19	5	7	7
**Other types of testicular tumor**	25	16	3	6
**Teratoma**	58	36	11	11
**Seminoma**	88	45	21	22
Data presented as numbers				

**Table 2:** A comparison of sperm concentration in different types of testicular cancer


**Diagnosis**	**S**	**T**	**M**	**O**
**Total number of cases**	88	58	19	25
**Number of patients with azoospermia**	4	2	1	3
**Mean sperm concentration in all of the patients (×10${}^{6}$/ml)**	22.4	23.4	30.2	21.5
**Minimum sperm concentration in non-azoospermic, patients (×10${}^{6}$/ml)**	0.09	0.1	1	0.2
**Highest sperm concentration in non-azoospermic, patients (×10${}^{6}$/ml)**	105	167	133	164
**Percentage of the patients with subnormal sperm concentration (< 20 × 10${}^{6}$/ml)**	60.2	62.1	57.9	68
**Percentage of the patients with azoospermia**	4.5	3.4	5	12
**Total number of non-azoospermic patients**	84	56	18	22
**Percentage of the patients with subnormal sperm concentration in non-azoospermic patients (< 20 × 10${}^{6}$/ml)**	58.3	60.7	55.6	63.6
**Mean of sperm concentration in non-azoospermic patients (×10${}^{6}$/ml)**	23.5	24.2	31.8	24.4
**Mean of sperm concentration in the patients with normal sperm concentration (×10${}^{6}$/ml)**	45.2	49.3	64.7	51.5
**Mean of sperm concentration in the patients with subnormal sperm concentration (×10${}^{6}$/ml)**	7.45	7.56	5.07	7.44
**Minimum sperm concentration in patients with normal sperm concentration (×10${}^{6}$/ml)**	20	20	31	20
S: Seminoma; T: Teratoma; M: Mixed germ cell tumor; O: Other types of testicular tumor				

**Table 3:** The mean of motility sluggish, non-progressive, and immotile in various types of testicular cancer


**Testicular cancer**	**Mean of sluggish motility**	**Mean of non-progressive motility**	**Mean of immotile**
**Seminoma**	13.68	5.74	42.63
**Teratoma**	12.59	7.07	42.22
**Mixed germ cell tumor**	12.05	3	41.89
**Other types of carcinoma**	11.28	3.32	38.96
Data presented as percentages			

## 4. Discussion 

Assessment of male infertility is based mainly on the standard semen analysis, which includes sperm count, motility, and sperm morphology. This study was undertaken with semen analyses of 190 patients who had been referred to the sub-fertility laboratory at the St Mary hospital, for semen banking. Based on the standard procedure recommended by WHO (1999), several aspects of their semen analyses were examined. The preliminary diagnosis in all of the cases was testicular tumor. Initial statistical analysis revealed that they were a suitable group for analysis as their age mean (29.75), median (29), and mode (28) were closely similar.

The cases were divided into four categories: seminoma, teratoma, mixed germ cell tumors and other types of tumor (i.e., embryonal carcinoma, yolk sac tumor). The results of the semen analyses were studied and categorized. The variables in this study were age, volume (in ml), population of sperm (million/ml), motility of sperm (assessed as excellent, sluggish, non-progressive or immotile), total sperm count (million/ejaculate), and morphology. These variables were used as their normal ranges have been specified by WHO.

In choosing a control group for comparison with the patients' results, consideration had to be given to the very important role played by the variable of age, because most patients with testicular carcinoma are young. Analysis of the data was in agreement with the finding of Gandini *et al.* (15), who found that the mean age of the seminoma patients differed from the mean age of patients with other types of testicular cancer and that the mean age of their seminoma patients was significantly higher than that of the groups of patients with embryonal carcinoma and mixed tumors. In contrast, Botchan *et al.* (16) found no mean age difference between patients with different types of testicular cancer which is not in agreement with the finding of this present study.

This significant difference suggests that the age range of the teratoma patients started earlier than that for seminoma patients and that the patients with the teratoma were usually younger than the patients with the seminoma. This finding was in agreement with some previous studies (16, 17). In the seminoma group, no patients was under 20 yr old, and 17 patients (19%) were older than 37 yr of age. The second significant difference was between the teratoma group and the volunteers. Dunnett *t* tests treated the volunteer group as the control group and compared all other groups against it. In this comparison, the teratoma group was the only group that differed significantly from the volunteers. In each group, the correlation of age and other variables was checked and only one significant correlation was found in the teratoma group. In this, the age was correlated with the total sperm count. In the teratoma group, as the patients got older, the total sperm counts were increased. This finding suggests that the effect of the teratoma in the patients of younger age could be more serious than in the older patients. Although, the concentration of the sperm and the volume of the semen did not show any significant correlation with age in the teratoma group, the correlation of the age and the total sperm counts indicates that both the volume of the ejaculate and concentration of the sperm were affected by the carcinoma and that this resulted in a significant correlation between the age and the total sperm counts (16).

Of the 190 patients, the mean semen volume was 2.6 ml, which according to the WHO reference range is an acceptable semen volume. This finding was in agreement with those of Panidis *et al.*, who found that there was no significant difference between the mean semen volume of their patients with testicular carcinoma and their control group of normal fertile men (18). Also, the findings of this present study are very similar to the findings of the studies of Bussen *et al.*, who found that the mean semen volume for their patients with testicular cancer was 2.5 ml and 2.8 ml, respectively (19).

Although the findings of Gandini *et al.* (15) were in agreement with the finding of this study for the semen volume, they showed no significant difference between the semen volumes for different types of testicular tumor. The finding of this study could suggest that even if the differences were not significant, there were some changes among the various groups.

The results of the semen analysis in this study showed that more than 61% of the patients with testicular tumor had an abnormal sperm concentration. Also, 5% of the patients were azoospermic. The mean of sperm concentration in all of the patients was 23.4 × 10${}^{6}$/ml and the mean sperm concentration in the patients with abnormal sperm concentration was 7.26 × 10${}^{6}$/ml. The findings of this study were in agreement with all the previous studies that suggest that testicular carcinoma diminishes the sperm concentration in patients (20, 21). Unfortunately, proteomic study showed defective cellular pathways in TC patients before cancer treatment (20).

Investigating each individual group of patients, based on their type of carcinoma, also showed that the mean of sperm concentration in those patients with abnormal sperm concentration, in each group, was significantly different from the value recommended by the WHO. This finding is consistent with the results of Petersen *et al.*, who concluded that there was a significant difference between the sperm count in patients with testicular carcinoma and healthy volunteers (21).

Although there were different effects among the various groups, the results showed that those patients diagnosed with “other types of tumor” such as embryonal carcinoma and yolk sac tumor had the most severe effects on their sperm concentration. Nevertheless, there was a clear finding that all types of testicular carcinoma studied could reduce the sperm concentration, as > 61% of the patients in this study had an abnormal sperm concentration. This finding is in agreement with the findings of two other studies that found that the semen quality in seminoma patients was better than that in the other types of testicular carcinoma (14, 15).

The results of the present study showed that > 56% of the patients had abnormal total sperm counts. The comparison between the various groups did not show any significant difference between them, which differs from the study by Gandini *et al.* (15), who found a significant difference between the total sperm counts in a seminoma group and a group of embryonal carcinoma.

All of the groups in the present study showed a significant difference between their total sperm count and that of the group of volunteers as a control. There was a significant difference between the mean total sperm counts of those patients with normal sperm counts.

The total sperm count is related to the sperm concentration and the volume of ejaculated semen. The result of this study showed that although the highest percentage of the patients with abnormal sperm concentration belonged to the patients diagnosed with “other types of tumor, while the seminoma group was ranked third, seminoma had the highest percentage for abnormal total sperm count (at 56%).

The mean of “excellent” sperm motility and “excellent plus sluggish” sperm motility were examined in all of the patients. Results showed that there were no significant differences between the groups. This does not agree with the finding of Gandini *et al.* (15), who found a significant difference between a seminoma group and an embryonal carcinoma group, but it is consistent with their further finding that there was no significant difference between the seminoma group and a group with mixed germ cell tumor and was also in agreement with their observation of no significant difference between their groups of mixed germ cell tumor and embryonal carcinoma.

The results showed that the mean “excellent” and “excellent plus sluggish” sperm motility, either for all of the patients together or in the individual groups, had a significant difference from the mean “excellent” and “excellent plus sluggish” sperm motility of the volunteers and from the values recommended by the WHO. This is in agreement the results of Botchan *et al.* (16), who found that there was a significant difference between the sperm motility of patients with testicular carcinoma and group of healthy volunteers. Also, the finding of the present study was in agreement with the results of the study of Panidis *et al.* (18) who found that 50% of the patients had abnormal sperm motility and their sperm motility was different from a group of fertile men as the control.

Comparison between the four different groups in the present study showed that the seminoma group had the lowest mean of “excellent” sperm motility and the patients with mixed germ cell tumor had the highest mean. All of these data suggest that patients with seminoma experienced the greatest effect of the carcinoma on their sperm motility. The findings of the present study differ from those of other studies by Gandini *et al.* (15) and Botchan *et al.* (16), which suggested that seminoma patients had better semen quality than patients with other testicular carcinoma.

Among 180 zoospermic patients, only three had nil “excellent” sperm motility and belonged to the seminoma group. This finding supports the suggestions that patients with seminoma had the worst value for “excellent” sperm motility and that this may be caused by the effects of the carcinoma. Sperm morphology is still one of the most controversial semen parameters in terms of its role in evaluating potential male fertility. As there have been very few previous studies that have explained the influence of testicular carcinoma on the morphology of sperm, the discussion that follows has to be based very largely on the findings of this present study.

The results of the present study show that the morphology of sperm is the most sensitive semen parameter that is affected by testicular carcinoma and this is in agreement with the similar finding of Panidis *et al.* (18). Of the 190 patients in the present study, who had various testicular carcinoma, only 15 had normal sperm morphology. Even these 15 patients with normal morphology had a mean morphology of 18.07%, which was significantly different from the lowest percentage of the morphology in the group of volunteers. This finding is in agreement with the study of Botchan *et al.* (16), who found that there was a significant difference between the normal sperm morphology in the patients with testicular cancer and healthy volunteers. The finding of Panidis *et al.* (18) was also in agreement with the finding of this study, as they found that 83.4% of their patients had abnormal sperm morphology and that there was a mean difference between their patients with testicular cancer and normal fertile men.

The most severe effects of testicular carcinoma on the morphology of sperm were seen, in the present study, in the group of patients diagnosed with “other types of tumor”, as all of the patients (100%) had abnormal sperm morphology with a mean morphology of 5.23%. This is in agreement with the finding of Gandini *et al.* (15), who showed that of the three groups of patients with testicular carcinoma, those patients with embryonal carcinoma had the lowest value of normal sperm morphology, being lower than that of the seminoma patients and patients with mixed germ cell tumors. In this latter group, three patients (12%) had too few sperm with normal morphology to count. The smallest effect of testicular carcinoma on sperm morphology was seen in the group of patients with mixed germ cell tumors, in which < 78% of the patients had abnormal sperm morphology, with a mean sperm morphology of 8.5%. None of the patients had too few normal sperm to count. All of these findings confirm that testicular carcinoma lead to alterations in sperm morphology and are in agreement with all of the limited number of previous studies (14, 16, 18, 21). Most carcinoma appeared to seriously impair sperm morphology in most cases.

## 5. Conclusion 

Impaired spermatogenesis is seen in most patients with testicular cancer before treatment with radiation, chemotherapy, or surgery. The causes of poor semen quality in cancer patients are not well-recognized, but the patients with impaired spermatogenesis should have precise examination to find out the correct diagnosis of problem and preserve the fertility before any treatment. Many mechanisms contribute to the impairment of semen, including the direct effect of the tumor on the testes and indirect effects such as hormones and secretions from the tumor, which should be considered on a case-by-case basis of male infertility. So, most carcinoma seriously impair sperm morphology. However, it is important to know the duration of problem, stage and grade of tumors, which might affect the results of evaluation. Future study should consider these limitations of the present study.

##  Conflict of Interest

The authors have no conflict of interest in this study.
